# Dietary Vitamin D_3_ Restriction Exacerbates Disease Pathophysiology in the Spinal Cord of the G93A Mouse Model of Amyotrophic Lateral Sclerosis

**DOI:** 10.1371/journal.pone.0126355

**Published:** 2015-05-28

**Authors:** Elnaz Moghimi, Jesse A. Solomon, Alexandro Gianforcaro, Mazen J. Hamadeh

**Affiliations:** 1 School of Kinesiology and Health Science, Faculty of Health, York University, Toronto, Ontario, Canada; 2 Muscle Health Research Centre, York University, Toronto, Ontario, Canada; University G. D'Annunzio, ITALY

## Abstract

**Background:**

Dietary vitamin D_3_ (D_3_) restriction reduces paw grip endurance and motor performance in G93A mice, and increases inflammation and apoptosis in the quadríceps of females. ALS, a neuromuscular disease, causes progressive degeneration of motor neurons in the brain and spinal cord.

**Objective:**

We analyzed the spinal cords of G93A mice following dietary D_3_ restriction at 2.5% the adequate intake (AI) for oxidative damage (4-HNE, 3-NY), antioxidant enzymes (SOD2, catalase, GPx1), inflammation (TNF-α, IL-6, IL-10), apoptosis (bax/bcl-2 ratio, cleaved/pro-caspase 3 ratio), neurotrophic factor (GDNF) and neuron count (ChAT, SMI-36/SMI-32 ratio).

**Methods:**

Beginning at age 25 d, 42 G93A mice were provided food *ad libitum* with either adequate (AI;1 IU D_3_/g feed; 12 M, 11 F) or deficient (DEF; 0.025 IU D_3_/g feed; 10 M, 9 F) D_3_. At age 113 d, the spinal cords were analyzed for protein content. Differences were considered significant at P ≤ 0.10, since this was a pilot study.

**Results:**

DEF mice had 16% higher 4-HNE (P = 0.056), 12% higher GPx1 (P = 0.057) and 23% higher Bax/Bcl2 ratio (P = 0.076) vs. AI. DEF females had 29% higher GPx1 (P = 0.001) and 22% higher IL-6 (P = 0.077) vs. AI females. DEF males had 23% higher 4-HNE (P = 0.066) and 18% lower SOD2 (P = 0.034) vs. AI males. DEF males had 27% lower SOD2 (P = 0.004), 17% lower GPx1 (P = 0.070), 29% lower IL-6 (P = 0.023) and 22% lower ChAT (P = 0.082) vs. DEF females.

**Conclusion:**

D_3_ deficiency exacerbates disease pathophysiology in the spinal cord of G93A mice, the exact mechanisms are sex-specific. This is in accord with our previous results in the *quadriceps*, as well as functional and disease outcomes.

## Introduction

Amyotrophic lateral sclerosis (ALS), also known as Lou Gehrig’s disease, is the most commonly occurring adult-onset motor neuron disease of unknown cause [1,2] and is typically diagnosed between 45 and 60 years of age [3,4]. It is characterized by degeneration of upper and lower motor neurons, resulting in skeletal muscle atrophy [5] and death by respiratory failure within 3–5 years of initial symptoms [6–8]. 90% of cases are of unknown etiology (sporadic ALS) [3,9], whereas the other 10% have inherited genetic mutations [3,10] (familial ALS), ~12% of these cases being a result of a mutation in the Cu^2+^/Zn^2+^ super-oxide dismutase 1 (SOD1) gene [11–14]. The most commonly used animal model of ALS is the G93A mouse model [15] that transgenically overexpresses the mutant SOD1 gene [10]. Their disease pathology and neurodegenerative patterns closely resemble that which is found in ALS patients [10]. On a cellular level, excessive stimulation of glutamate receptors [16] leads to a large influx of calcium ion into the post synaptic neuron, resulting in a destructive cascade of membrane, cytoplasmic and nuclear events [17]. These include oxidative damage [18,19], oxidative stress [20,21], inflammation [22], compromised neurotrophic factor release [22] and apoptosis [13].

Some nutrition-based interventions have shown effectiveness in mitigating ALS disease severity in animal models of ALS [23]. Vitamin D is a fat-soluble vitamin with hormone-like properties that is essential for health, growth and development [24]. Vitamin D_3_ and/or its metabolites [calcidiol (25(OH)D_3_) and calcitriol (1,25(OH)_2_D_3_)] can protect dopaminergic neurons against the neurotoxic effects of glutamate and dopaminergic toxins [25], and has anti-inflammatory and modulatory effects on CNS components such as neurotrophins and growth factors [26]. Vitamin D treatment can improve compromised functional outcomes and muscle physiology in humans and rodents, whereas vitamin D receptor (VDR) knockout mice have loss of motor function and muscle mass [27]. Vitamin D reduces the expression of biomarkers associated with oxidative stress and inflammation in diseases that share common pathophysiologies with ALS. Vitamin D deficiency has been associated with the development of inflammatory and immune diseases such as type II diabetes [28], multiple sclerosis [29], dementia and Alzheimer’s disease [30]. A deficiency in vitamin D reduces the amount of calcium buffering protein, thus leading to higher lipid peroxidation and protein damage [31]. When investigating the effects of vitamin D on biomarkers of oxidative stress in obese children aged 7–14 y, obese children with 25(OH)D insufficiency (serum calcidiol <50 nmol/L) had significantly elevated 3-nitrotyrosine (3-NY) levels, a marker of protein damage, vs. non-deficient obese children (serum calcidiol >50 nmol/L) [32]. A partial correlation analysis showed an inverse relationship between 25(OH)D and 3-NY (r = -0.424, P = 0.001).

A retrospective study in ALS patients found that those with serum calcidiol levels <25 nmol/L increased their death rate by 6 fold and their rate of decline by 4 times, and were associated with a marked shorter life expectancy compared to patients with serum calcidiol levels >75 nmol/L [33]. We have previously demonstrated the detrimental effects of vitamin D_3_ restriction in the G93A mouse model of ALS [34–37]. Dietary vitamin D_3_ at 2.5% the adequate intake (AI) resulted in lower paw grip endurance (PaGE) and motor performance [37], and in the *quadriceps* of female G93A resulted in increased inflammation [35] and apoptosis [36], when compared to their AI counterparts.

Does vitamin D_3_ restriction directly impact the CNS? And, if it does, will vitamin D_3_ deficiency explain the functional outcomes in our previous study [37]. Hence, the objective of this study was to investigate the effects of vitamin D deficiency via dietary restriction (0.025 IU/g feed) vs. adequate intake (1 IU/g feed) on oxidative damage, antioxidant capacity, inflammation, apoptosis, neurotrophic factor and neuron count in the spinal cord of the G93A transgenic mouse model of ALS

## Methods

### Ethical Statement

The experimental protocol used in this study followed the guidelines of the Canadian Council of Animal Care and was approved by York University Animal Research Ethics Board (protocol # 2007–9). All the necessary steps were taken to minimize suffering and distress to the mice in the study.

### Animals

Male B6SJL-TgN(SOD1-G93A)1Gur hemizygous mice (No. 002726) were harem-bred with non-affected female B6SJL control mice (No. 100012; Jackson Laboratory, Bar Harbor, ME). We identified the presence of the human-derived G93A transgene by using polymerase chain reaction (PCR) amplification of DNA extracted from ear tissue as outlined by Sigma-Aldrich (XNAT REDExtract-N-Amp Tissue PCR Kit; XNAT-1KT). All breeding mice were housed 3 females per 1 male, and consumed Research Diet AIN-93G (1 IU D_3_/g feed; Research Diet, New Brunswick, NJ). All animals were housed individually at age 25 d in a 12 h light/dark cycle.

### Study Design

42 (22 M, 20 F) G93A mice consumed a diet that contained an adequate intake of vitamin D_3_ (1 IU/g feed; Research Diet AIN-93G; Product # D10012G; Research Diets Inc, New Brunswick NJ [38]) *ad libitum* after weaning (21 d). At age 25 d, the mice were individually caged and divided into one of two groups: 1) adequate vitamin D_3_ (AI; 1 IU D_3_/g feed; 12 M, 11 F; Research Diet AIN-93G) or 2) deficient vitamin D_3_ (DEF; 1/40 IU D_3_/g feed; 10 M 9 F; Product #D10030801; Research Diets Inc, New Brunswick, NJ) (Table 1).

**Table 1:** Nutrient content of the adequate intake (AI) and deficient (DEF) vitamin D_3_ diets.

Nutrient	Diet	
	AI	DEF
**Energy (kcal/g)**	4	4
**Carbohydrate (%)**	64	64
**Protein (%)**	20	20
**Fat (%)**	7	7
**Vitamin D**_**3**_ **(IU/g)**	1^a^	0.025^b^
**Calcium (%)**	0.5^c^	0.5^c^
**Vitamin mix V10037 (mg/g)**	10	10
**Mineral mix S100022G (mg/g)**	35	35

Diets provided by Research Diets (based on AIN-93G; New Brunswick, NJ; AI product # D10012G; HiD product # D08080101;).

^a^, included in vitamin mix V10037

^b^, included in vitamin mix V13203 [110]

^c^, included in mineral mix S100022G [111]. Table adopted from Solomon *et al*, PLoS ONE 2011 [37].

When the mice reached a clinical score (CS; disease severity) of 3.0, food and calorie-free gel (Harlan-Gel, Harlan Teklad, Madison WI) were placed on the floor of the cage to fulfill ethics requirements. Endpoint was determined as previously described by Solomon *et al* 2011 [38]. The calorie-free gel contained synthetic polymers (WATER LOCK superabsorbent polymer G-400, G-430, G-500, G-530; 95% by weight) and methanol (4.5% by weight). Two researchers who were blinded to the diets conducted all measurements.

### Tissue Collection

At age 113 d, mice were sacrificed and spinal cords were harvested. The mice were placed and kept under anesthesia with gaseous isoflurane as the tissue was collected and placed in individual sterile polyethylene tubes for immediate freezing in liquid nitrogen. Samples were stored at -80°C.

### Spinal Cord Homogenization

Spinal cords were weighed, and minced with a glass-Teflon Port-Evenhejm homogenizer (5% wt/vol) in radioimmunoprecipitation assay (RIPA) buffer (1:20) containing 50 mM tris HCL 8.0 (Bioshop, TRS002.500, Burlington, Ontario), 150 mM NaCl (BioBasic Canada, 7647145, Markham, Ontario), 0.1% SDS (Bioshop, SDS001.500, Burlington, Ontario), 0.5% sodium deoxycholate (Bioship, DCA333.50, Burlington, Ontario), 1% NP-40 (Thermo Scientific, 28324, Rockford, Illinois), 5 mM EDTA pH 8.0 (Bioshop, EDT001.500, Burlington, Ontario) and 1 mM PMSF (Sigma-Aldrich, 93482, St. Louis, Missouri). The protease inhibitor cocktail (Roche, 11836153001, Manheim, Germany) was added to the buffer in accordance to manufacturer’s instructions (1:100) prior to homogenization. Mouse spinal cord was homogenized for about 40 grinds using constant force to ensure consistency and homogeneity of samples. Homogenates were divided in roughly equal volumes in eppendorf tubes and were placed on a shaker at 4°C for 30 minutes. The homogenates were then centrifuged at (600 *g*) for 20 min at 4°C. The resulting supernatant was decanted, put into newly labeled eppendorf tubes and immediately stored at -80°C. The protein concentration was determined using the BCA Protein Assay technique [39]. The supernatant concentration was measured at 562 nm using an ultraviolet spectrophotometer (Cecil 9200 Super Aquarius, Cambridge, UK). Protein concentrations were presented as mg/ml.

## Western Blot

Equal amounts of protein were size-separated by 12.5% sodium dodecyl sulfate-polyacrylamide gel electrophoresis (SDS-PAGE) and were transferred to nitrocellulose membranes (#165–3322, Bio-Rad Mini-PROTEAN 2 electrophoresis system, Mississauga, ON, Canada) at 100 V for 2 h. The membranes were blocked in 3% fat free milk (SMI-36), 5% fat free milk (SOD2, catalase, TNF-α, IL-6) or 5% BSA (4-HNE, 3-NY, GPx1, IL-10, Bax, Bcl-2, pro-caspase 3, cleaved caspase 3, GDNF, ChAT, SMI-32) diluted in Tris-buffered saline with tween (1%) for 2 h at room temperature and incubated with primary antibodies in 3% fat free milk (SMI36), 5% fat free milk (catalase, TNF- α, IL-6), 1% BSA (SOD2, cleaved caspase 3, GNDF, ChAT), 3% BSA (IL-10, SMI32) or 5% BSA (4-HNE, 3-NY, GPx1, Bax, Bcl-2, pro-caspase 3) against 4-HNE (1:800; Abcam, ab46545), 3-NY (1:1000; Abcam, ab110282), SOD2 (1:8000; Abcam, ab13533), catalase (1:3500; Abcam, ab1877-10), GPx1 (1:800; Abcam, 22604), TNF- α (1:2000; Abcam, ab9739) IL-6 (1:1000; Abcam, ab6672), IL-10 (1:2000; Abcam, ab9969), Bax (1:1000; Cell Signaling Technology, 2772), Bcl-2 (1:1000; Cell Signaling Technology, 2870), pro-caspase 3 (1:1000; Millipore, 04–440), cleaved caspase 3 (1:1000; Millipore, 04–439), GDNF (1:1000; Abcam, a18956), ChAT (1:1000; Abcam, ab85609), SMI-32 (1:1000; Abcam, ab28029) and SMI-36 (1:1000; Abcam, ab24572), overnight at 4°C. Each antibody and its corresponding anti-GAPDH set were loaded on a separate gel. Equal loading was verified by ponceau staining, as well as probing for glyceraldehyde 3-phosphate dehydrogenase (GAPDH; 1:100,000; MAB374, Millipore). The antigen-antibody complexes were detected by incubating the membranes in anti-rabbit (1: 5000; Novus Biologicals, NB730-H) or anti-mouse (1:5000; Novus Biologicals, NB7539) HRP conjugated secondary antibodies at room temperature for 2 h in 3% fat free milk (SMI36), 5% fat free milk (4-HNE, catalase, TNF- α, IL-6), 1% BSA (SOD2, cleaved caspase 3, GDNF, ChAT), 3% BSA (IL-10, SMI32) or 5% BSA (3-NY, GPx1, Bax, Bcl-2, pro-caspase 3). Immunoreactive proteins were visualized with enhanced chemiluminescence (sc-2048, Santa Cruz Biotechnology), and scanned using Kodak Imaging Station 4000MM Pro (Carestream Health, Inc. Rochester, NY, USA). Protein intensity was standardized to GAPDH and analyzed using Carestream MI (v 5.0.2.30, NY, USA). Representative western blot bands for the biomarkers are found in S1 Fig.

### Calculations

Human equivalent dosage (HED) was calculated according to the US FDA [40]:

$$HED\;=\;Animal\;dose\;(mg/kg)\times \;{\left[animal\;weight\;\left(kg\right)÷human\;weight\;\left(kg\right)\right]}^{0.33}$$

### Statistical analysis

We established planned comparisons between DEF vs. AI. A one-tailed independent t-test was used to determine differences between the diets within each sex, because we hypothesized *a priori* that absolute and body weight-adjusted spinal cord weight, antioxidant activity, neurotrophic factors and neuronal count would be lower in DEF vs. AI; whereas oxidative stress and apoptosis would be higher in DEF vs. AI. These are based on studies conducted by us and other researchers [23,27,32,34–38,41–45]. A Student's t-test was used to determine diet and sex differences in absolute and body weight-adjusted *tibialis anterior*, *quadriceps* and brain weights. All statistical analyses were completed using GraphPad Prism 6 for Macintosh (GraphPad Software Inc, La Jolla, CA). Data were presented as means ± standard error of mean (SEM). Significance was set to P ≤ 0.10, since this was a pilot study.

## Results

### Oxidative Damage

#### 4-HNE

**Fig 1:**
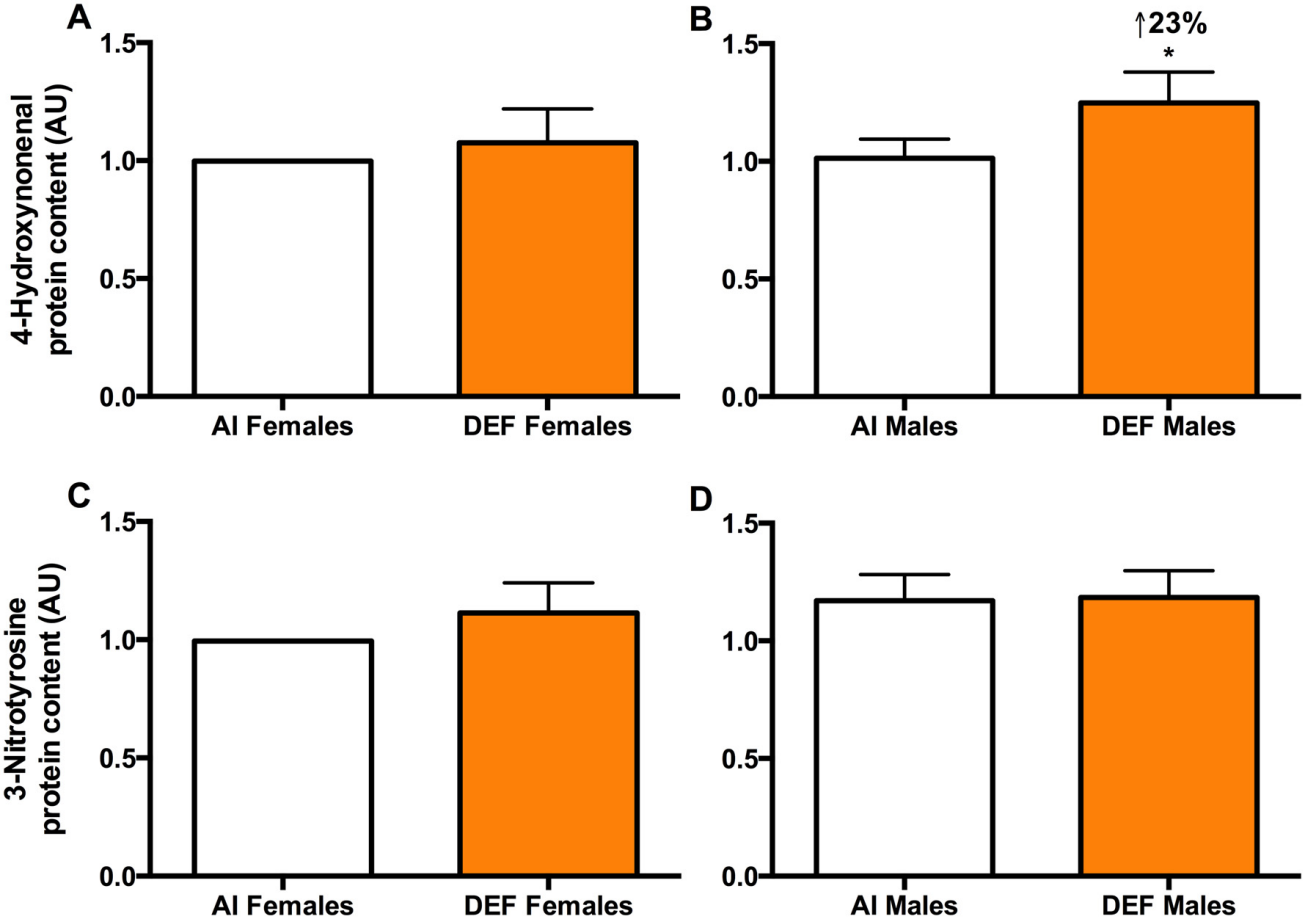
Oxidative damage in DEF vs. AI G93A mice. 4-HNE (A and B) and 3-NY (C and D) protein content (arbitrary units; AU) in spinal cord of 42 G93A mice: 23 adequate vitamin D_3_ intake (AI; 1 IU D_3_/g feed; 12 M, 11 F) and 19 deficient vitamin D_3_ intake (DEF; 0.025 IU D_3_/g feed; 10 M, 9 F). 4-*Hydroxynonenal (4-HNE*, *A and B)*: DEF mice had 16% higher 4-HNE protein content vs. AI (P = 0.056). DEF males had 23% higher 4-HNE protein content vs. AI males (P = 0.066). *3-Nitrotyrosine (3-NY*, *C and D)*: There was no significant difference in 3-NY protein content between the diets. AI males had 18% higher 3-NY protein content vs. AI females (P = 0.073). Data presented as means ± SEM.

DEF mice had 16% higher 4-HNE protein content vs. AI (P = 0.056). DEF males had 23% higher 4-HNE protein content vs. AI males (P = 0.066) (Fig 1B).

#### 3-NY

There was no significant difference in 3-NY protein content between the diets (Fig 1C and 1D). AI males had 18% higher 3-NY protein content vs. AI females (P = 0.073). Data presented as means ± SEM.

### Antioxidant Enzymes

#### SOD2

**Fig 2:**
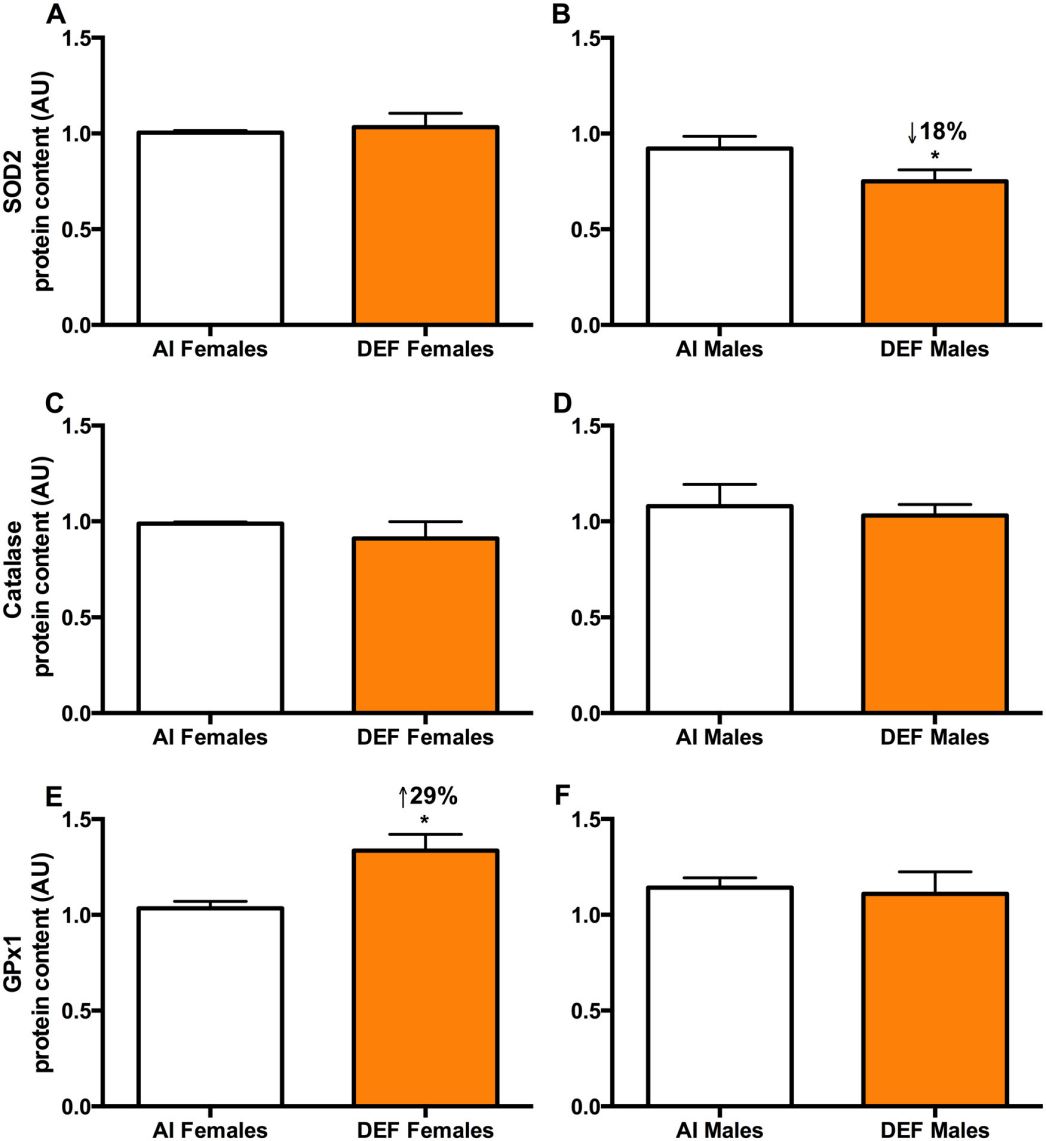
Antioxidant enzymes in DEF vs. AI G93A mice. SOD2 (A and B), catalase (C and D) and GPx1 (E and F) protein content (arbitrary units; AU) in spinal cord of 42 G93A mice: 23 adequate vitamin D_3_ intake (AI; 1 IU D_3_/g feed; 12 M, 11 F) and 19 deficient vitamin D_3_ intake (DEF; 0.025 IU D_3_/g feed; 10 M, 9 F). *SOD2 (A and B)*: DEF males had 18% lower SOD2 protein content vs. AI males (P = 0.034). DEF males had 27% lower SOD2 protein content vs. DEF females (P = 0.004). *Catalase (C and D)*: There was no significant difference in catalase protein content between the diets or between the sexes. *GPx1 (E and F)*: DEF mice had 12% higher GPx1 protein content vs. AI (P = 0.057). DEF females had 29% higher GPx1 protein content vs. AI females (P = 0.001). AI males had 10% higher GPx1 protein content vs. AI females (P = 0.054). DEF males had 17% lower GPx1 protein content vs. DEF females (P = 0.070). Data presented as means ± SEM.

DEF males had 18% lower SOD2 protein content vs. AI males (P = 0.034) (Fig 2B). DEF males had 27% lower SOD2 protein content vs. DEF females (P = 0.004).

#### Catalase

There was no significant difference in catalase protein content between the diets or between the sexes (Fig 2C and 2D)

#### GPx1

DEF mice had 12% higher GPx1 protein content vs. AI (P = 0.057). DEF females had 29% higher GPx1 protein content vs. AI females (P = 0.001) (Fig 2E). AI males had 10% higher GPx1 protein content vs. AI females (P = 0.054). DEF males had 17% lower GPx1 protein content vs. DEF females (P = 0.070).

### Inflammation

#### TNF- α

**Fig 3:**
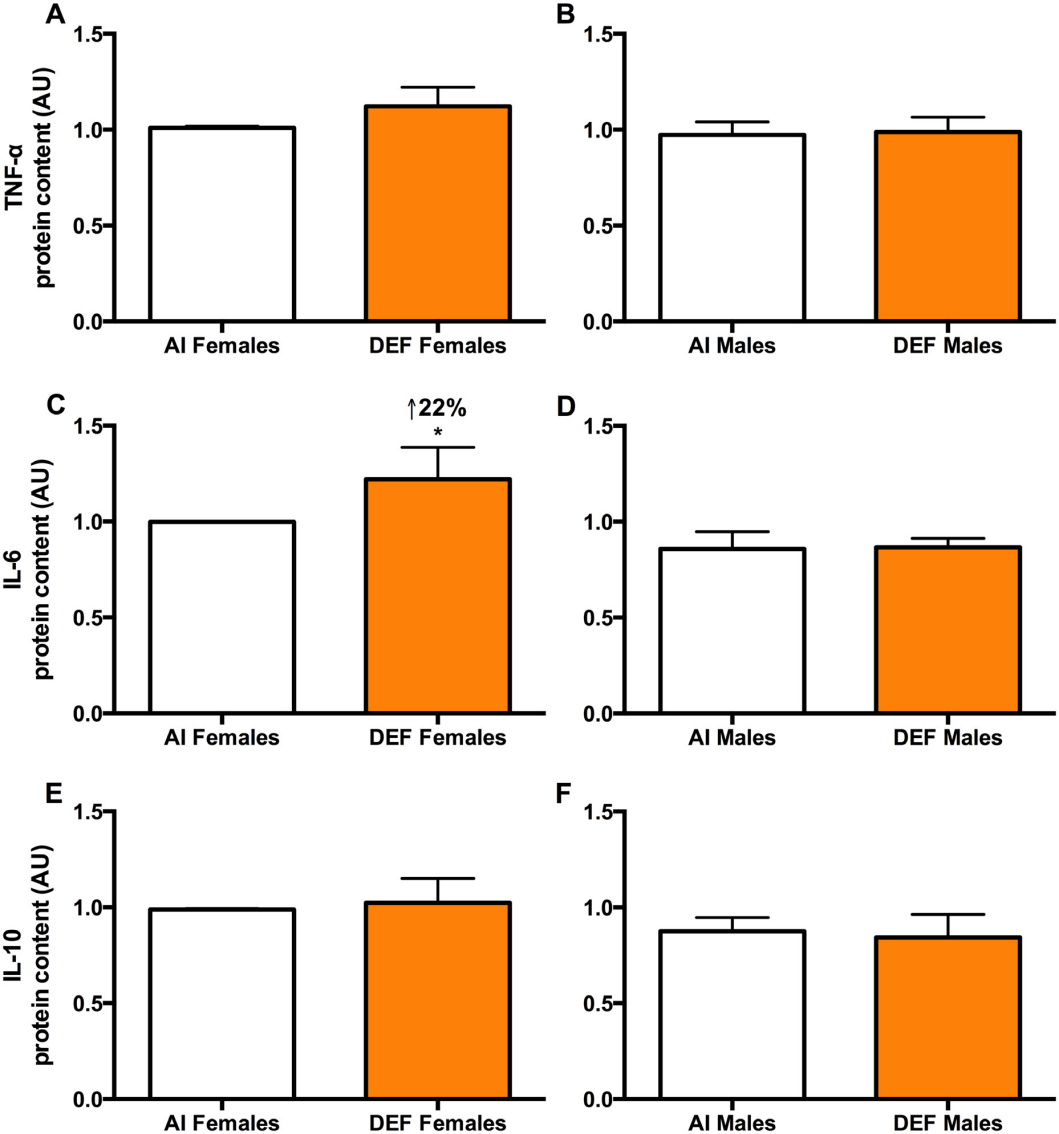
Inflammation in DEF vs. AI G93A mice. TNF- α (A and B), IL-6 (C and D) and IL-10 (E and F) protein content (arbitrary units; AU) in spinal cord of 42 G93A mice: 23 adequate vitamin D_3_ intake (AI; 1 IU D_3_/g feed; 12 M, 11 F) and 19 deficient vitamin D_3_ intake (DEF; 0.025 IU D_3_/g feed; 10 M, 9 F). *TNF-α (A and B)*: There was no significant difference in TNF-α protein content between the diets or between the sexes. *IL-6 (C and D)*: DEF females had 22% higher IL-6 protein content vs. AI females (P = 0.077). AI males had 14% lower IL-6 protein content vs. AI females (P = 0.075). DEF males had 29% lower IL-6 protein content vs. DEF females (P = 0.023). *IL-10 (E and F)*: There was no significant difference in IL-10 protein content between the diets. AI males had 11% lower IL-10 protein content vs. AI females (P = 0.074). Data presented as means ± SEM.

There was no significant difference in TNF-α protein content between the diets or between the sexes (Fig 3A and 3B).

#### Il-6

DEF females had 22% higher IL-6 protein content vs. AI females (P = 0.077) (Fig 3C). AI males had 14% lower IL-6 protein content vs. AI females (P = 0.075). DEF males had 29% lower IL-6 protein content vs. DEF females (P = 0.023).

#### IL-10

There was no significant difference in IL-10 protein content between the diets (Fig 3E and 3F). AI males had 11% lower IL-10 protein content vs. AI females (P = 0.074).

### Apoptosis

#### Bax

**Fig 4:**
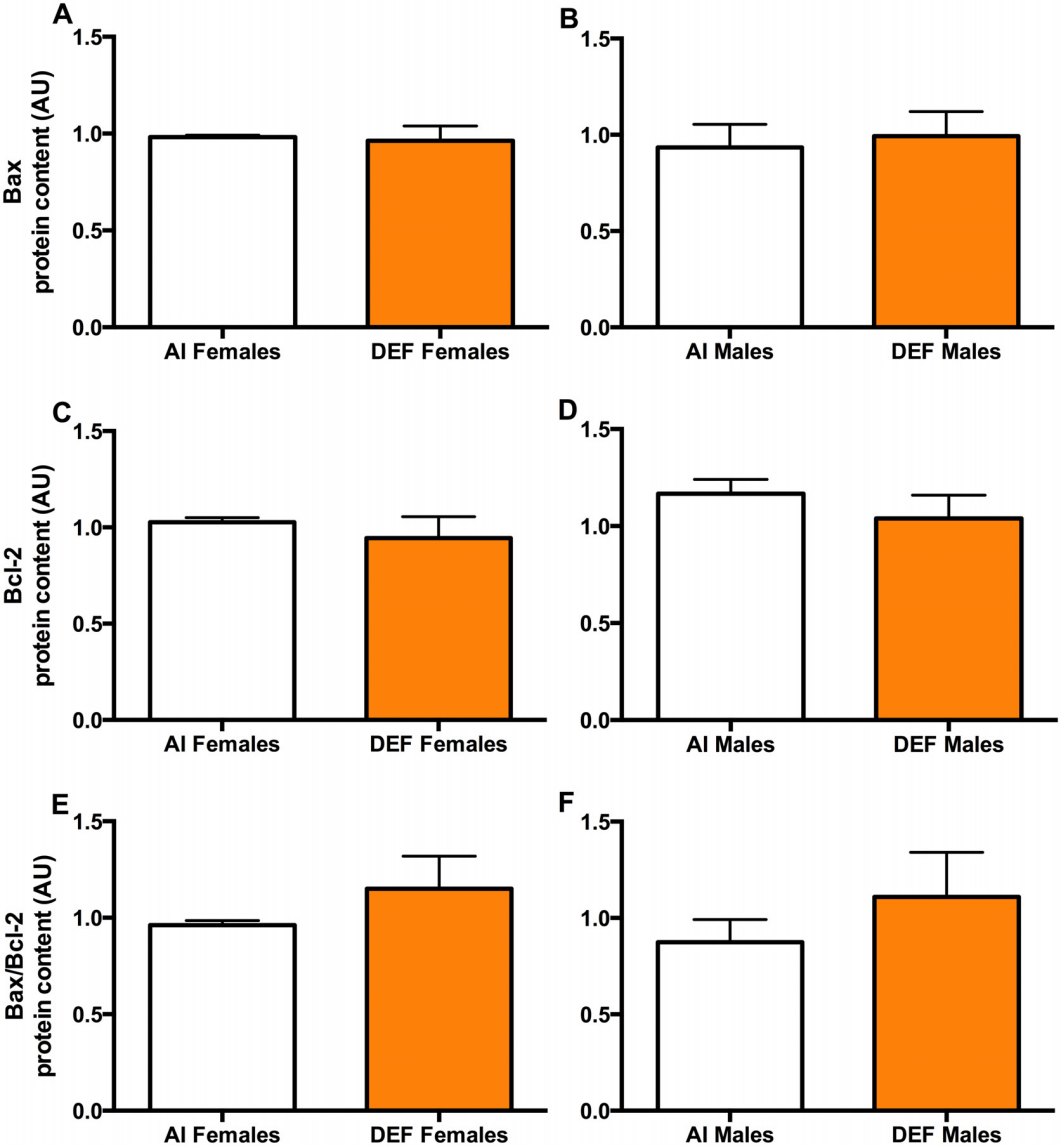
Bax and Bcl-2 in DEF vs. AI G93A mice. Bax (A and B), Bcl-2 (C and D) and Bax/Bcl-2 ratio (E and F) protein content (arbitrary units; AU) in spinal cord of 42 G93A mice: 23 adequate vitamin D_3_ intake (AI; 1 IU D_3_/g feed; 12 M, 11 F) and 19 deficient vitamin D_3_ intake (DEF; 0.025 IU D_3_/g feed; 10 M, 9 F). *Bax (A and B)*: There was no significant difference in Bax protein content between the diets or between the sexes. *Bcl-2 (C and D)*: There was no significant difference in Bcl-2 protein content between the diets. AI males had 14% higher Bcl-2 protein content vs. AI females (P = 0.048). *Bax/Bcl-2 ratio (E and F)*: DEF mice had 23% higher Bax/Bcl-2 protein content vs. AI (P = 0.076). Data presented as means ± SEM.

There was no significant difference in Bax protein content between the diets or between the sexes (Fig 4A and 4B).

#### Bcl-2

There was no significant difference in Bcl-2 protein content between the diets (Fig 4C and 4D). AI males had 14% higher Bcl-2 protein content vs. AI females (P = 0.048).

#### Bax/Bcl-2 ratio

DEF mice had 23% higher Bax/Bcl-2 protein content vs. AI (P = 0.076).

#### Pro-caspase 3

**Fig 5:**
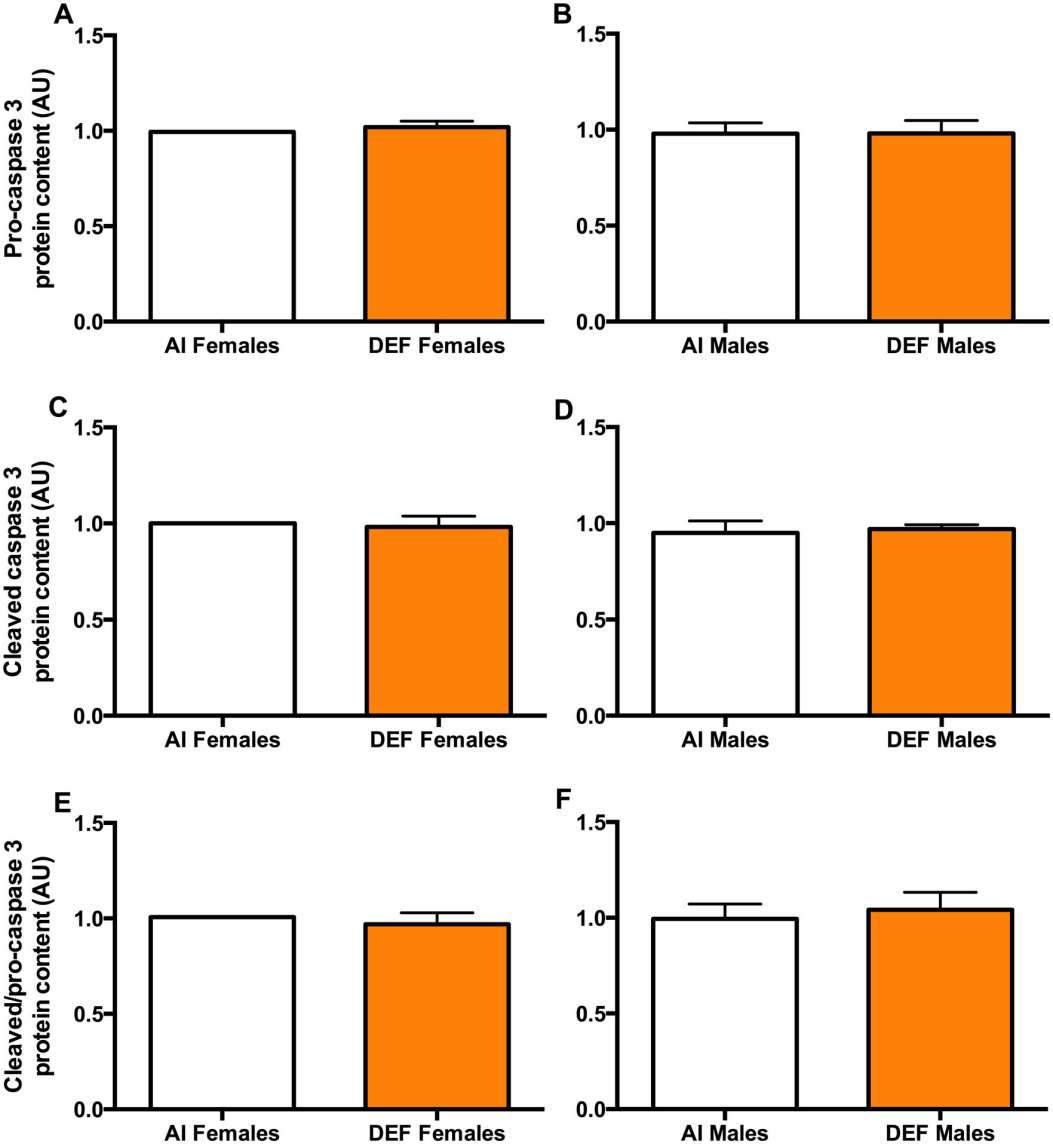
Caspase 3 in DEF vs. AI G93A mice. Pro-caspase 3 (A and B), cleaved caspase 3 (C and D) and cleaved/pro-caspase 3 (E and F) protein content (arbitrary units; AU) in spinal cord of 42 G93A mice: 23 adequate vitamin D_3_ intake (AI; 1 IU D_3_/g feed; 12 M, 11 F) and 19 deficient vitamin D_3_ intake (DEF; 0.025 IU D_3_/g feed; 10 M, 9 F). There was no significant difference in pro-caspase 3, cleaved caspase 3 and cleaved/pro-caspase 3 protein content between the diets or between the sexes. Data presented as means ± SEM.

There was no significant difference in pro-caspase protein content between the diets or between the sexes (Fig 5A and 5B).

#### Cleaved caspase 3

There was no significant difference in cleaved caspase 3 protein content between the diets or between the sexes (Fig 5C and 5D).

#### Cleaved/pro-caspase 3

There was no significant difference in cleaved/pro-caspase 3 protein content between the diets or between the sexes (Fig 5E and 5F).

### Neurotrophic Factor

#### GDNF

**Fig 6:**
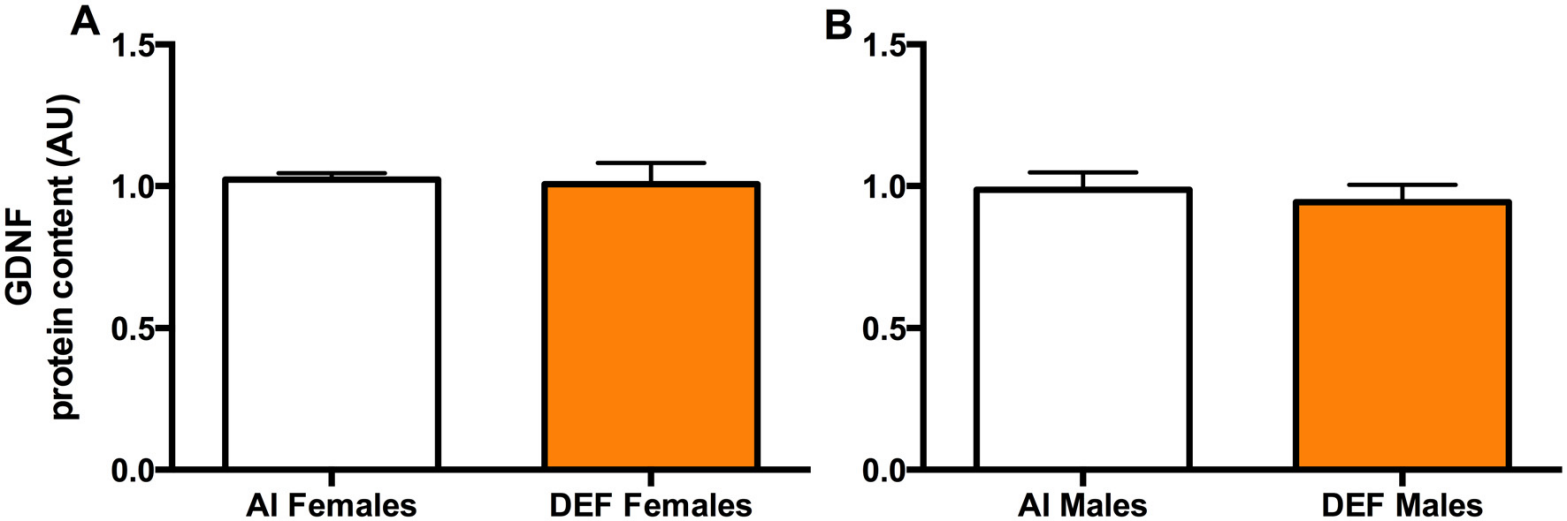
Neurotrophic factor in DEF vs. AI G93A mice. GDNF protein content (A and B) (arbitrary units; AU) in spinal cord of 42 G93A mice: 23 adequate vitamin D_3_ intake (AI; 1 IU D_3_/g feed; 12 M, 11 F) and 19 deficient vitamin D_3_ intake (DEF; 0.025 IU D_3_/g feed; 10 M, 9 F). There was no significant difference in GDNF protein content between the diets or between the sexes. Data presented as means ± SEM.

There was no significant difference in GDNF protein content between the diets or between the sexes (Fig 6A and 6B).

### Neuron Count

#### ChAT

**Fig 7:**
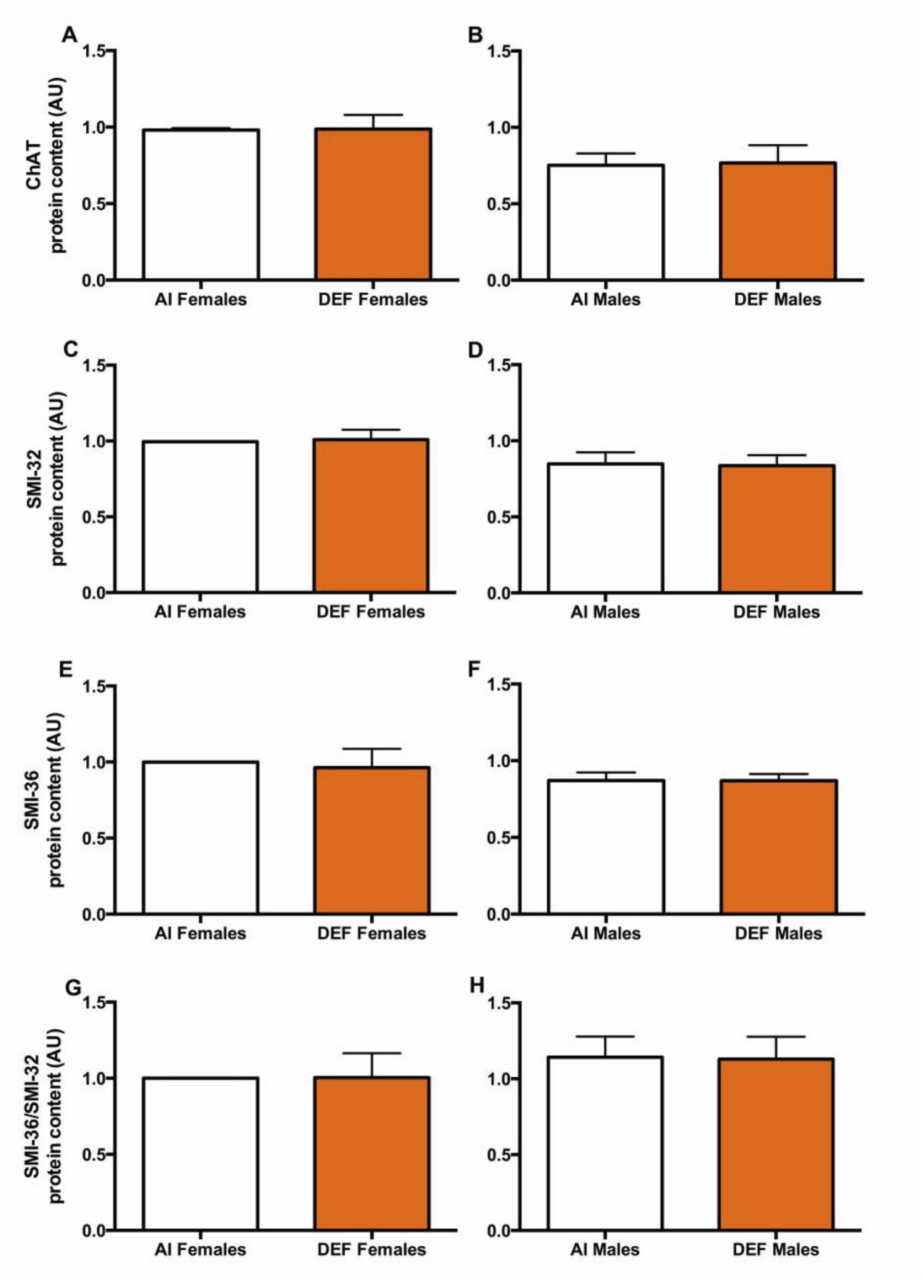
Neuron count in DEF vs. AI G93A mice. ChAT (A and B), SMI-32 (C and D), SMI-36 (E and F) and SMI-36/SMI-32 ratio (G and H) protein content (arbitrary units; AU) in spinal cord of 42 G93A mice: 23 adequate vitamin D_3_ intake (AI; 1 IU D_3_/g feed; 12 M, 11 F) and 19 deficient vitamin D_3_ intake (DEF; 0.025 IU D_3_/g feed; 10 M, 9 F). *ChAT (A and B)*: There was no significant difference in ChAT protein content between the diets. AI males had 23% lower ChAT protein content vs. AI females (P = 0.005). DEF males had 22% lower ChAT protein content vs. DEF females (P = 0.082). *SMI-32 (C and D)*: There was no significant difference in SMI-32 protein content between the diets. AI males had 15% lower SMI-32 protein content vs. AI females (P = 0.039). DEF males had 17% lower SMI-32 protein content vs. DEF females (P = 0.046). *SMI-36 (E and F)*: There was no significant difference in SMI-36 protein content between the diets. AI males had 13% lower SMI-36 protein content vs. AI females (P = 0.016). *SMI-36/SMI-32 ratio (G and H)*: There was no significant difference in SMI-36/SMI-32 protein content between the diets or between the sexes. Data presented as means ± SEM.

There was no significant difference in ChAT protein content between the diets (Fig 7A and 7B). AI males had 23% lower ChAT protein content vs. AI females (P = 0.005). DEF males had 22% lower ChAT protein content vs. DEF females (P = 0.082)

#### SMI-32

There was no significant difference in SMI-32 protein content between the diets (Fig 7C and 7D). AI males had 15% lower SMI-32 protein content vs. AI females (P = 0.039). DEF males had 17% lower SMI-32 protein content vs. DEF females (P = 0.046).

#### SMI-36

There was no significant difference in SMI-36 protein content between the diets (Fig 7E and 7F). AI males had 13% lower SMI-36 protein content vs. AI females (P = 0.016).

#### SMI-36/SMI-32

There was no significant difference in SMI-36/SMI-32 protein content between the diets or between the sexes (Fig 7G and 7H).

### Spinal cord weights

**Fig 8:**
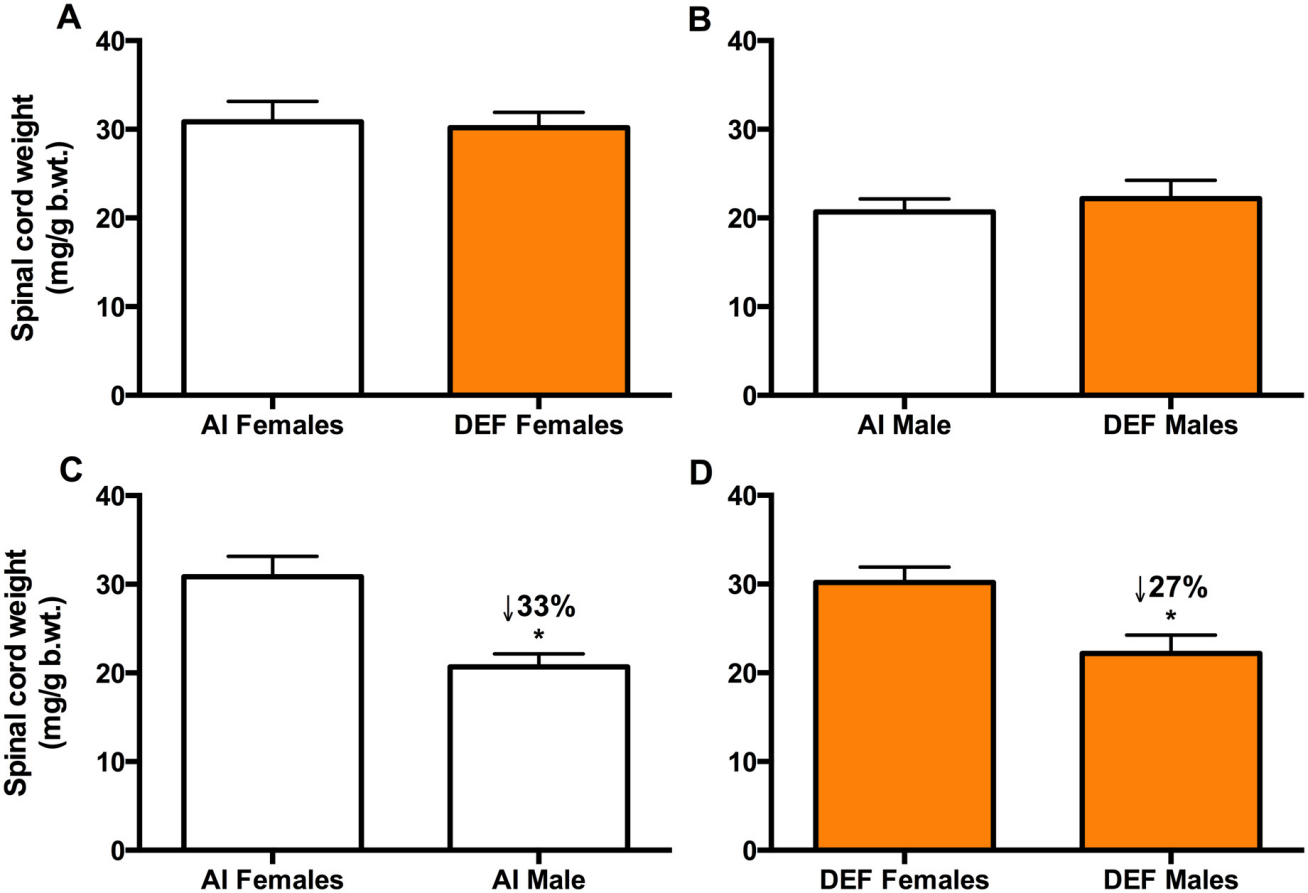
Body weight-adjusted spinal cord weights. Body weight-adjusted spinal cord weight (mg/g b.wt.) of 42 G93A mice: 23 adequate vitamin D_3_ intake (AI; 1 IU D_3_/g feed; 12 M, 11 F) and 19 deficient vitamin D_3_ intake (DEF; 0.025 IU D_3_/g feed; 10 M, 9 F). *Between the diets (A and B)*: There was no significant difference in body weight-adjusted spinal cord weights between the diets. *Between the sexes (C and D)*: AI males had 33% lighter body weight-adjusted spinal cord weight vs. AI females (P = 0.001), and DEF males had 27% lighter body weight-adjusted spinal cord weight vs. DEF females (P = 0.005). Data presented as means ± SEM.

Absolute spinal cord weight was not different between the diets (Table 2). Between the sexes, AI males had 15% lighter absolute spinal cord weight vs. AI females (P = 0.065), and DEF males had 16% lighter absolute spinal cord weight vs. DEF females (P = 0.053) (Table 2). There was no significant difference in body weight-adjusted spinal cord weights between the diets (Table 2; Fig 8A and 8B). Between the sexes, AI males had 33% lighter body weight-adjusted spinal cord weight vs. AI females (P = 0.001) (Table 2; Fig 8C), and DEF males had 27% lighter body weight-adjusted spinal cord weight vs. DEF females (P = 0.005) (Table 2; Fig 8D).

**Table 2:** Spinal cord weight between the diets and sexes.

Spinal cord weights	Females			Males			Within-AI between-sex differences	Within-DEF between-sex differences
	AI	DEF	P value	AI	DEF	P value	P value	P value
**Absolute spinal cord weight (mg)**	583±43	581±33	NS	494±37	489±42	NS	P = 0.065	P = 0.053
**Body weight-adjusted spinal cord weight (mg/g b.wt.)**	31±2	30±5	NS	21±2	22±7	NS	P = 0.001	P = 0.005

Data are means ± SEM. AI, adequate intake; DEF, deficient vitamin D. AI Males, n = 12; AI Females, n = 11. DEF Males, n = 10; DEF Females, n = 9

## Discussion

We investigated the effects of vitamin D deficiency, via dietary vitamin D_3_ restriction equivalent to 2.5% the rodent AI, on markers of oxidative damage, antioxidant enzymes, inflammation, apoptosis, growth factors and neuron count in the spinal cord of G93A mice, a rodent model of ALS. Dietary vitamin D restriction at 1/40^th^ AI exacerbates disease pathophysiology as DEF mice displayed higher levels of lipid peroxidation and apoptosis compared to AI. DEF females had higher inflammation and a compensatory increase in the antioxidant GPx1 compared to AI females. Conversely, DEF males had reduced antioxidant capacity compared to AI males. When comparing differences between the sexes, DEF males had lower antioxidant enzymes and neuronal count compared to DEF females. The extant sexual dimorphism both in AI and DEF mice confirms that though detrimental, vitamin D deficiency negatively impacts different pathways depending on the sex, having a more deleterious effect in males compared to females.

Sexual dimorphism was observed in our current study as vitamin D deficiency caused differential results in males vs. females. Sexual dimorphism exists in a multitude of neurological and mental disorders such as multiple sclerosis (MS), Alzheimer’s disease (AD), Parkinson’s disease (PD) and ALS [27]. ALS is predominant in males, but with increasing age the ratio of male-to-female diagnoses becomes smaller. The sex difference may be a result of different aromatase activity, the enzyme that converts testosterone into estradiol. This activity is neuroprotective [46,47] and is higher in cortical female astrocytes than cortical male astrocytes [48]. The dimorphic nature of the enzyme can protect astrocytes, as well as other CNS cell types such as neurons, from damage in females. It has also been postulated that the presence of the sex hormone estrogen plays a role in this dimorphism. Recent clinical evidence has shown that estrogen treatment reduces the risk and delays the onset of many neurodegenerative diseases [49]. In primary cultures of Wistar rat spinal cord, estradiol exerts neuroprotective effects *in vivo* [50]. *In vitro*, estradiol protects cerebral neurons against glutamate excitotoxicity [50]. Administration of the phytoestrogen genistein to male mSOD1 mice reduces the difference in disease onset and mortality between the sexes (prior to genistein administration, disease onset and mortality were reached sooner in males vs. females), confirming the strong role of sex hormones [51]. Ovariectomy of G93A mice accelerates disease progression, and a high-dose of 17β-estradiol significantly slows down disease progression in these mice [52] In the presence of vitamin D_3_, estrogen synthesis is increased [53], allowing both estrogen and vitamin D_3_ to exert neuroprotective effects. A confirmed synergy exists between vitamin D_3_ and estrogen, which is also found in the spinal cord [54]. Estrogen causes estrogen receptor-mediated down-regulation of CYP24A1 (calcitriol deactivating enzyme) transcription to increase net calcitriol concentration, and thus enhance vitamin D function [54]. As well, estrogen up-regulates VDR to enhance vitamin D potency, and, in turn, calcitriol causes VDR-mediated up-regulation of estrogen synthase to enhance endogenous estrogen synthesis [55]. As a result, basal CNS calcitriol levels are higher in females vs. males [56].

A deficiency in vitamin D reduces the amount of calcium buffering protein, thus leading to higher lipid peroxidation [31]. This could explain why DEF males had 23% higher 4-HNE protein content, a marker of oxidative damage, vs. AI males. This difference was not observed in DEF females vs. AI. This is in agreement with estrogen’s protective role. Co-exposure of 17β-estradiol and 4-HNE in PC12 cell lines showed that estrogen was significantly effective against the cytotoxic response of 4-HNE [57]. This is because 17β-estradiol has the ability to stabilize mitochondrial potential against oxidative stress [58]. As well, estrogen works similarly to vitamin D to establish cellular calcium homeostasis. Chronic 17β-estradiol treatment represses glutamate receptor-mediated Ca^2+^ influx [59]. In ALS, a disruption of calcium transport can form free radicals that cause lipid peroxidation in the cell [60]. Vitamin D induces the synthesis of proteins such as parvalbumin that help maintain cellular calcium homeostasis [61], thus lowering lipid peroxidation, in diabetic rats [62]. Vitamin D also reduces malondialdehyde (MDA), a marker of lipid peroxidation, by stimulating the gene expression of calcium buffering proteins calbindin-D28k and calbindin-d9k [31]. Obese children deficient in vitamin D (serum calcidiol <50 nmol/L) had higher lipid peroxidation as marked by increased 3-NY and MDA levels compared to non-deficient obese children (serum calcidiol >50 nmol/L) [32].

The protective role of estrogen is also a factor in why AI males had 18% higher 3-NY protein content vs. AI females. 17β-estradiol’s antioxidative effect reduces 3-NY immunoreactivity [63]. Brain cell cultures of mice exposed to 17β-estradiol significantly reduced 3-NY levels regardless of whether or not they have been exposed to superoxide [64]. Estrogen can directly inhibit nitric oxide synthase (NOS) activity, thereby reducing peroxynitrite and subsequently 3-NY generation [65].

Vitamin D deficiency also had a negative impact on antioxidant capacity. SOD2 was 18% lower in DEF males vs. AI males. Compared to healthy individuals, SOD2 activity is lower in the brain and spinal cord of ALS patients [66]. It is normally expected that in response to higher oxidative damage, antioxidant capacity increases. Thus, the exact mechanism that leads to a reduction in SOD2 activity is not well understood. It is possible that the loss of activity could be a result of post-translational modification. During CNS injury, nitric oxide (NO) is released at a high rate, which reacts with superoxide and leads to the production of nitrogenous species such as peroxynitrite (ONOO^-^) [67]. Peroxynitrite, which is highly prevalent in ALS spinal cord, is the only known biological oxidant to inactivate enzymatic activity, nitrate important tyrosine residues and cause dityrosine formation in SOD2 [68]. Higher levels of peroxynitrite lead to increased production of 3-NY [69]. Compared to their female counterparts, AI and DEF males had 18% and 6% higher 3-NY levels, respectively, which could explain why SOD2 was lower in DEF males, but not DEF females, vs. AI. A reduction in SOD2 in DEF males may be related to vitamin D’s impact on nuclear factor-kappa B (NF-κB). In ALS, activated microglia use the NF-κB pathway to induce mitochondrial dysfunction inhibition of SOD2 and motor neuron death [70,71]. NF-κB leads to mitochondrial dysfunction inhibition of SOD2 through nitration, by activating inducible NOS (iNOS) [72]. The local conversion of calcidiol to calcitriol in the CNS is a neuroprotective response that inhibits NF-κB-related iNOS induction [73]. Without the protective effects of vitamin D, this neuroprotection diminishes, which may explain the lower levels of SOD2 in DEF males.

GPx1 was 29% higher in DEF females vs. AI females. Female hypertensive Wister rats have shown increased GPx1 activity and lower reduced glutathione (GSH) levels [74]. Under physiological conditions, vitamin D has an inverse relationship with GPx1 activity and a positive association with glutathione reductase (GR) activity [75]. This relationship is due to GSH’s role in maintaining intracellular redox balance. Increasing the activity of GR and decreasing GPx1 function allow vitamin D to enhance the GSH pool. In vitamin D deficiency however, excessive inflammation, as reflected by high IL-6 levels, increases GPx1 activity as a means of reducing oxidative protein injury [76]. This also explains why, in this study, DEF females did not have a significant increase in 3-NY vs. AI. Vitamin D deficiency increased IL-6 levels by 22% compared to AI females, thereby elevating GPx1 protein content by 29%. Thus, the adaptive increase in GPx1 may indicate heightened inflammation and cellular damage. In many cancers, including esophageal cancer, GPx1 further induces malignancy and promotes tumor progression, effects that can be reduced by vitamin D [77]. In breast cancer patients, high expression of GPx1 was associated with high rate of patient mortality and shorter overall survival [78], which may be due to NF-κB. When bound to the promoter region of GPx1, NF-κB upregulates its function and expression upstream [77]. Vitamin D and VDR inhibit NF-κB expression and thus decrease GPx1 levels [79]. In females, estrogen’s ability to convert calcidiol to calcitriol [80] can heighten the ability of the vitamin to inhibit the NF-κB pathway, thereby reducing GPx1 levels. This may explain why AI males had 10% higher GPx1 protein content than AI females.

IL-6 levels were 22% higher in DEF females vs. AI females. Damage to the CNS causes an upregulation in IL-6 and other pro-inflammatory cytokines such as TNF-α [81]. IL-6 plays a great role in astrocyte and microglia activation, microglial proliferation as well as gliosis [82]. Though it is meant to repair, gliosis can work as a double-edged sword: it can produce neurotrophic factors and protect the CNS from toxins, but it can also produce neurotoxins such as nitric oxide, an important factor in free-radical genesis [82]. Estrogen is known to regulate IL-6 expression in different cell types [83]. In biliary epithelial cells, estrogen had the ability to stimulate IL-6 production in both the neoplasmic and non-neoplasmic cells that expressed estrogen receptor alpha [83]. A study on the effects of gonadal steroids on IL-6 in peripheral blood mononuclear cells showed that 17β-estradiol promotes IL-6 production and release [84]. As well, deficiency in vitamin D after trauma puts women at a greater risk of elevated IL-6 levels [85]. This is in accord with Miller *et al*’s study that showed that women with serum 25(OH)D levels of <37.5 nmol/L at the time of hip fracture had higher serum IL-6 levels in the year after the hip fracture [85]. Alternatively, testosterone maintains low IL-6 levels [86–88]. This explains why we observed 14% lower IL-6 in AI males vs. AI females, and 29% lower IL-6 in DEF males vs. DEF females. Interestingly, testosterone is also negatively associated with TNF-α [88], whereas estradiol increases its expression [89]. AI and DEF males had non-significant 4% and 12% lower TNF-α levels vs. their female counterparts. Higher TNF-α levels in DEF females reflects vitamin D’s impact on this inflammatory cytokine. In females, a significant inverse association exists between 25(OH)D and TNF-α, whereby vitamin D deficiency increases levels of the inflammatory cytokine [90]. A study in endurance-trained athletes showed that circulating TNF-α does not increase linearly with decreasing 25(OH)D concentration. Instead, it is abruptly higher in those that are vitamin D deficient (lower than 80 nmol/L) [91]. In neuron and glial cells, matrix metalloproteinase-9 (MMP-9) regulates TNF-α levels and is found in high levels in damaged ALS motor neurons [92]. MMPs are largely associated with inflammation and work to remodel and break down the extracellular matrix and regulate leukocyte migration within it. Calcitriol reduces MMP-9 activity, thereby reducing TNF-α levels [93]. Within the spinal cord of G93A mice, upregulation of the p38 mitogen activated protein kinase (p38MAPK), a signaling pathway responsible for cell death, is associated with the upregulation of TNF-α receptors [94]. Calcitriol reduces p38MAPK activity, thereby reducing TNF- α levels [95,96]. In terms of anti-inflammatory cytokines, AI males had 11% lower IL-10 protein content vs. AI females. IL-10 has been shown to increase in the presence of estrogen [97]. Malaria infected female mice were shown to have higher IL-10 levels compared to males [98]. In terms of its relationship with inflammatory cytokines, higher IL-6 levels induce IL-10 production [99]. This explains why the higher IL-10 levels we observed in females were commensurate with higher IL-6 protein content.

There was 23% higher Bax/Bcl-2 in DEF mice vs AI mice. However, this may not necessarily indicate that apoptosis was prevalent. This is because no changes in activated caspase 3 and neuron count were observed between the diets. Though Bax/Bcl-2 ratio was elevated in DEF mice, the increase was not sufficient enough to activate caspase 3, the effector molecule in the apoptotic pathway. High Bax/Bcl-2 ratio increases the vulnerability of neurons to apoptosis [100], and is observed in neuromuscular disorders such as ALS [101]. What this may indicate is that apoptotic proteins can reduce neuronal viability without leading to large-scale apoptosis. This is also confirmed in our previous study in HiD female *quadriceps* that had reached the threshold of vitamin D_3_ toxicity. A 242% increase in Bax/Bcl-2 [102] only corresponded to an 87% increase in cleaved/pro-caspase 3 [43]. Thus, a deficiency in vitamin D may increase the susceptibility of motor neurons to apoptosis without necessarily leading to large-scale apoptosis. Vitamin D has been shown to reduce pro-apoptotic (Bax) and increase anti-apoptotic proteins (Bcl-2) [103]. A reduction in calcium buffering capacity brought about by vitamin D deficiency may cause the cell to exert calcium-induced excitotoxicity, which leads to elevated levels of Bax/Bcl-2.

With respect to neuron count, both AI and DEF males had lower ChAT (23% and 22%, respectively) and SMI-32 (15% and 17%, respectively) compared to their female counterparts, likely due to the protective effect of estrogen in females. In mSOD1 mice, onset, disease progression and survival are dependent on sex; males lose body weight more rapidly following disease onset and die sooner than females [104,105]. A reduction in body weight reflects muscle atrophy brought about by motor neuron degeneration. As with increased motor neuron count in females, it is possible that damaged motor neurons are also more prevalent. This explains why SMI-36 levels were 13% lower in AI males vs. AI females. This can also explain why there was no difference in SMI-36/SMI-32 ratio between AI males and AI females, as SMI-32 and SMI-36 levels were both lower in males vs. females. Ultimately, neuron count was not different between the diets, indicating that even though vitamin D deficiency may exacerbate disease pathophysiology, it does not have an impact on neuron count.

On a tissue level, there was no significant difference in body weight-adjusted spinal cord weights between the diets. However, a sexual dimorphism was confirmed as AI males and DEF males had 33% and 27% lighter body weight-adjusted spinal cord weight vs. their female counterparts. This may be due to the protective effects of estrogen in the female spinal cord. These results contrast our previous study that found no difference in body weight-adjusted brain weights between the diets and sexes [45]. Correlational analysis showed that there was no association between body weight-adjusted brain weights [45] and body weight-adjusted spinal cord weights. This confirms that ALS pathology within the CNS is mainly localized to the spinal cord.

This study outlines the detrimental effects of vitamin D deficiency and confirms the lower paw grip endurance and motor performance that was observed in our previous study in the same mouse model [37]. When compared to AI mice, DEF mice had 25% lower paw grip endurance (PaGE) AUC and 19% lower motor performance. Between the sexes, AI males had lower ability to move, PaGE and motor performance compared to AI females. AI males also had a higher clinical score, hastened disease onset, and reached hind limb paralysis and endpoint faster compared to AI females. The current study confirms that the functional outcomes observed are linked to neuronal damage in the spinal cord. Previous studies on spinal cord injury (SCI) rats have shown that motor performance disturbance following SCI is associated with the severity of spinal cord pathology [106]. Damage in the spinal cord also reflects that in the *quadriceps*, where DEF female, but not male, G93A mice had higher inflammation and apoptosis as compared to AI females [35,36]. Despite the fact that the spinal cords of DEF females had higher inflammation, DEF male spinal cords were more susceptible to damage as marked by lower levels of SOD2 and neuron count. We postulate that this is due to the protective effects of estrogen in females. The restricted vitamin D_3_ intake in this study corresponds to ~25 IU/d for an 80 kg man and ~20 IU/d for a 70 kg woman. These values may be insufficient for patients with ALS. Indeed, Karam *et al*’s study on ALS patients found that supplementation with 2000 IU of vitamin D_3_/day for 9 months improved ALS functional rating scale score [107]. A retrospective study on ALS patients found that those with serum calcidiol levels <25 nmol/L increased their death rate by 6 fold and their rate of decline by 4 times, and were associated with a marked shorter life expectancy compared to patients with serum calcidiol levels >75 nmol/L [33]. Furthermore, Guamanian Chamorros with ALS have serum calcitriol levels in the low to low-normal range [108]. Based on these studies, and given our previous studies supplementing ALS mice with 10x and 50x AI, we restate our previous hypothesis that the optimal therapeutic vitamin D dosage, both functionally and cellularly, lies between 10x and 50x AI vitamin D [27,44,45].

In conclusion, the present study demonstrates that vitamin D deficiency exacerbates disease pathophysiology in the G93A mouse model of ALS. This is marked by increased inflammation and oxidative damage and lower antioxidant capacity. However, it is important to note that sexual dimorphism exists and that the pathways that vitamin D deficiency negatively impacts differ between males and females [109].

## Supporting Information

S1 Fig Western blot representative bands for markers of oxidative damage, antioxidant enzymes, inflammation, apoptosis, neurotrophic factor and neuron damage. Representative immunoblots of 4-HNE, 3-NY, SOD2, catalase, GPx1, TNF-α, IL-6, IL-10, Bax, Bcl-2, pro-caspase 3, cleaved caspase 3, GDNF, ChAT, SMI-32 and SMI-36 protein expression in the spinal cord of 42 G93A mice: 23 adequate vitamin D_3_ intake (AI; 1 IU D_3_/g feed; 12 M, 11 F) and 19 deficient vitamin D_3_ intake (DEF; 0.025 IU D_3_/g feed; 10 M, 9 F). Each antibody and its corresponding anti-GAPDH set were loaded on a separate gel. Protein intensity was standardized to GAPDH.
(TIFF)
