# Supplementary figures and images for: Dietary Vitamin D3 Restriction Exacerbates Disease Pathophysiology in the Spinal Cord of the G93A Mouse Model of Amyotrophic Lateral Sclerosis
Source: PLoS One. 2015 May 28;10(5):e0126355. doi: 10.1371/journal.pone.0126355 (PMC4447353; doi:10.1371/journal.pone.0126355)

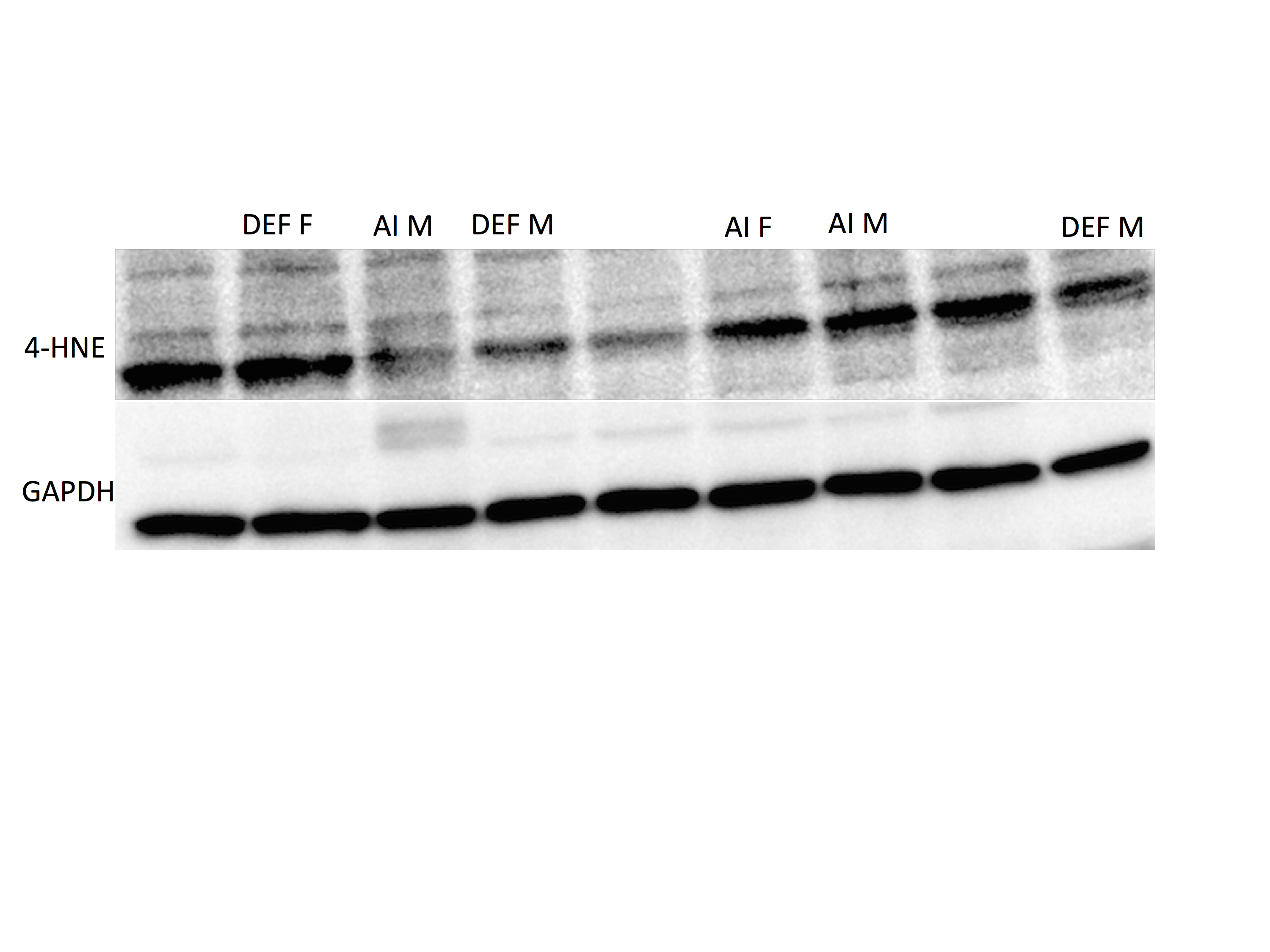

Supplement: S1 Fig — Representative immunoblots of 4-HNE, 3-NY, SOD2, catalase, GPx1, TNF-α, IL-6, IL-10, Bax, Bcl-2, pro-caspase 3, cleaved caspase 3, GDNF, ChAT, SMI-32 and SMI-36 protein expression in the spinal cord of 42 G93A mice: 23 adequate vitamin D3 intake (AI; 1 IU D3/g feed; 12 M, 11 F) and 19 deficient vitamin D3 intake (DEF; 0.025 IU D3/g feed; 10 M, 9 F). Each antibody and its corresponding anti-GAPDH set were loaded on a separate gel. Protein intensity was standardized to GAPDH. (TIFF) [file pone.0126355.s001.tiff]
